# Distinct Roles of MicroRNA-1 and -499 in Ventricular Specification and Functional Maturation of Human Embryonic Stem Cell-Derived Cardiomyocytes

**DOI:** 10.1371/journal.pone.0027417

**Published:** 2011-11-16

**Authors:** Ji-Dong Fu, Stephanie N. Rushing, Deborah K. Lieu, Camie W. Chan, Chi-Wing Kong, Lin Geng, Kitchener D. Wilson, Nipavan Chiamvimonvat, Kenneth R. Boheler, Joseph C. Wu, Gordon Keller, Roger J. Hajjar, Ronald A. Li

**Affiliations:** 1 University of California School of Medicine, Davis, California, United States of America; 2 Center of Cardiovascular Research, Mount Sinai School of Medicine, New York, New York, United States of America; 3 Department of Medicine, The University of Hong Kong, Hong Kong; 4 Department of Anatomy, The University of Hong Kong, Hong Kong; 5 Department of Physiology, The University of Hong Kong, Hong Kong; 6 Stem Cell and Regenerative Medicine Consortium, The University of Hong Kong, Hong Kong; 7 Heart, Brain, Hormone and Healthy Aging Research Center, LKS Faculty of Medicine, The University of Hong Kong, Hong Kong; 8 Departments of Medicine and Radiology, Stanford University, Palo Alto, California, United States of America; 9 Laboratory of Cardiovascular Science, National Institute on Aging, National Institutes of Health, Baltimore, Maryland, United States of America; 10 McEwen Central for Regenerative Medicine, University Health Network, Toronto, Ontario, Canada; Brigham & Women's Hospital - Harvard Medical School, United States of America

## Abstract

**Background:**

MicroRNAs (miRs) negatively regulate transcription and are important determinants of normal heart development and heart failure pathogenesis. Despite the significant knowledge gained in mouse studies, their *functional* roles in *human* (h) heart remain elusive.

**Methods and Results:**

We hypothesized that miRs that figure prominently in cardiac differentiation are differentially expressed in differentiating, developing, and terminally mature human cardiomyocytes (CMs). As a first step, we mapped the miR profiles of human (h) embryonic stem cells (ESCs), hESC-derived (hE), fetal (hF) and adult (hA) ventricular (V) CMs. 63 miRs were differentially expressed between hESCs and hE-VCMs. Of these, 29, including the miR-302 and -371/372/373 clusters, were associated with pluripotency and uniquely expressed in hESCs. Of the remaining miRs differentially expressed in hE-VCMs, 23 continued to express highly in hF- and hA-VCMs, with miR-1, -133, and -499 displaying the largest fold differences; others such as miR-let-7a, -let-7b, -26b, -125a and -143 were non-cardiac specific. Functionally, LV-miR-499 transduction of hESC-derived cardiovascular progenitors significantly increased the yield of hE-VCMs (to 72% from 48% of control; p<0.05) and contractile protein expression without affecting their electrophysiological properties (p>0.05). By contrast, LV-miR-1 transduction did not bias the yield (p>0.05) but decreased APD and hyperpolarized RMP/MDP in hE-VCMs due to increased I_to_, I_Ks_ and I_Kr_, and decreased I_f_ (p<0.05) as signs of functional maturation. Also, LV-miR-1 but not -499 augmented the immature Ca^2+^ transient amplitude and kinetics. Molecular pathway analyses were performed for further insights.

**Conclusion:**

We conclude that miR-1 and -499 play differential roles in cardiac differentiation of hESCs in a context-dependent fashion. While miR-499 promotes ventricular specification of hESCs, miR-1 serves to facilitate electrophysiological maturation.

## Introduction

MicroRNAs (miRs) are non-encoding RNAs of ∼22 nucleotides that function as negative transcriptional regulators via degradation or inhibition by RNA interference [1], [2]. To date, 706 human miRs have been identified, of which approximately 50% of pre-miR sequences are located within introns according to the Sanger Database. In mouse models, several miRs have been implicated in normal cardiovascular development (e.g., miR-1, 18b, 20b, 21, 106a, 126, 133, 138, and 208) [3]–[8]. For instance, a 50% decrease in total miR-1 results in embryonic death attributable to ventricular septal defects and cardiac dysfunction [5]; whereas, miR-1 over-expression in adult murine ventricular (V) cardiomyocytes (CMs) promotes arrhythmogenesis by slowing conduction and depolarizing the sarcolemmal membrane via post-transcriptional repression of the Kir2.1-encoded inwardly rectifying current (I_K1_) and connexin (Cx) 43-mediated gap junction [9]. In hypertrophic adult rat VCMs, down-regulation of miR-1/miR-133 levels promotes automaticity via up-regulation of HCN2/HCN4, but this defect can be reversed by forced expression of miR-1/miR-133 [10], [11]. Various recent profiling efforts have further revealed profound alterations of miR expression in the pathogenesis of human heart failure [12]–[14]. Despite the knowledge gained from these studies, however, the functional roles of miRs in human cardiogenesis remains elusive due to the paucity of cause-and-effect data in human heart cells. Given that significant species differences are known to exist (e.g., miRs that regulate pluripotency genes were discovered in mouse but many have no known human homologs [15]), further studies of miRs in human heart cells are therefore warranted. Using self-renewable, pluripotent human (h) embryonic stem cells (ESCs) [16], along with primary human fetal (hF) and adult (hA) heart cells, we uncovered miRs that figure prominently in cardiac specification and development. Specifically, our experiments demonstrate that miR-499 promotes ventricular specification while miR-1 serves to facilitate their electrophysiological maturation.

## Materials and Methods

### Cell Culture and Cardiac differentiation

The HES2 (ESI, Singapore) hESC line was maintained on irradiated mouse embryonic fibroblasts (MEF) at 37°C with 5% CO_2_. The culture medium consisted of DMEM/F12 (Invitrogen) supplemented (all from Invitrogen) with 15% knockout serum, 1% non-essential amino acids, 1 mmol/L L-glutamine, 0.1 mmol/L β-mercaptoethanol, and basic fibroblast growth factor of 20 ng/ml. Specification of human tripotent KDR^low^/c-kit^neg^ cardiovascular progenitors into CMs using the activin, BMP-4 and VEGF induction was as reported [17]. In brief, hESCs were suspension-cultured to form cardiogenic embryoid bodies (EBs) or “cardiospheres” under serum- and feeder-free conditions. At day 1, the aggregates were incubated in media containing Activin A, and BMP-4, at 37°C under low (5%) oxygen for 4 days. At day 4, Activin A and BMP-4 were removed, followed by replenishing with media containing hDKK1 and VEGF. At day 8, cardiospheres were provided with fresh media, and fed again at day 12, supplied with bFGF and incubated at 37°C under normal ambient oxygen conditions. Fresh media was supplied every 3–4 days. Cardiac derivatives typically appeared after 12∼14 days. The yield of hESC-derived CMs with this method of directed cardiac differentiation is typically ∼50% as gauged by troponin T expression [17] (vs. <<1% by the conventional method of EB formation [18] as specified in some of the experiments).

### Isolation of hESC-derived CMs and Lentivirus (LV)-mediated gene transfer

Human ESC-derived CMs are known to contain a heterogenous population of ventricular, atrial and pacemaker derivatives [18]–[20]. For profiling experiments, ventricular (V) hESC-CMs were purified to avoid ambiguities due to the presence of contaminating non-ventricular CMs and non-cardiac cells. To accomplish this, post-differentiation 20-day old cardiospheres were transduced by recombinant LV-MLC2v-mCherry particles at a titer of >10^6^ and an MOI of 3. The efficacy of using the myosin light chain-2v promoter (MLC2v) to drive the expression of a reporter protein for identifying ventricular CMs, as we recently reported [21], was first confirmed by two independent methods: 1) Figure S1 shows that only human fetal left ventricular CMs, but not HEK293, cells expressed mCherry after LV-MLC2v-mCherry transduction, indicating the cardiac specificity of our construct. 2) Upon transduction of hESC-CMs by LV-MLC2v-mCherry, fluorescent cells typically appeared 72 hours later and could be FACS-sorted (BD FACSAria™ II). Electrophysiologically, mCherry^+^ hESC-derived CMs displayed ventricular action potentials (AP), exclusively (n = 15). FACS-sorted ventricular hESC-CMs were immediately used for miR/RNA extraction.

Single hESC-CMs were isolated for electrophysiological experiments as previously described [20], [22], [23] Beating outgrowths were microsurgically dissected from HES2-derived cardiospheres (20∼24 days) by a glass knife, followed by incubation in collagenase II solution (1 mg/ml) at 37°C for 30 min. The isolated cells were incubated with KB solution containing (mM): 85 KCl, 30 K_2_HPO_4_, 5 MgSO_4_, 1 EGTA, 2 Na_2_-ATP, 5 pyruvic acid, 5 creatine, 20 taurine, 20 D-glucose, at room temperature for 30 min. After the cells were plated on laminin-coated glass coverslips for 1 hr at 37°C, the culture media was added carefully and refreshed the next day. For miR overexpression, LV-CMV-miR-X-EF1α-GFP (System Biosciences, Mountain View, CA), or LV-miR-X™ for short, was incubated overnight, followed by replacing with fresh media. Positively transduced cells were identified by GFP and used for experiments 4 to 7 days after transduction. For controls, the identical backbone described above without any miR coding sequence (i.e. LV-CMV-BLANK-EF1α-GFP or LV-BLANK) or an antagomir (LV-CMV-anti-X-EF1α-GFP or LV-anti-X) as designed and recommended by System Bioscience was used as indicated. Specific miR overexpression of the experimental groups was confirmed by qPCR.

### Isolation of human fetal and adult ventricular CMs

Human fetal (hF) and adult (hA) left ventricular (V) CMs were isolated and experimented according to protocols approved by the UC Davis IUPAC and IRB (Protocol #200614787-1 and # 200614594-1). Briefly, fetal human hearts (16–18 weeks, Advanced Bioscience Resources, INC. Alameda, CA) and adult human hearts (18+ years, National Disease Research Interchange, Philadelphia, PA) were perfused with enzymatic solutions using a customized Langendorff apparatus as we previously described [24].

### MiR and Transcriptomic Profiling

Cell samples were suspended and lysed in Trizol (Invitrogen). After adding 1∶4 volume chloroform, aqueous and organic phases were separated using heavy PLG tubes (Eppendorf). MiRs was extracted from the aqueous phase using the miRNeasy kit (Qiagen, Valencia, CA), and stored at −80°C before use. Microfluidic uParaflow chips (Atactic Technologies, Houston TX) containing probes for Sanger microRNA Database v11.0 were used to profile miR expression; Sentrix WG-6 beadchips (Illumina, San Diego, CA) were used to profile mRNA expression. Microarray data were analyzed using the WebArrayDB for miRs (Sidney Kimmel Cancer Center, San Diego, CA) and BeadStudio for transcriptomic (Illumina) software packages [25]. Expression was normalized using background subtraction and cubic spline (BeadStudio) or composite LOESS normalization (WebArrayDB). Differential expressions in microarrays were assessed using the Analysis of Variance test (ANOVA) and results with p-value <0.05 were considered to be significant.

### Electrophysiological characterization

Electrophysiological experiments were performed using the whole-cell patch-clamp technique with an Axopatch 200B amplifier and the pClamp9.2 software (Axon Instruments Inc., Foster City, CA) [26], [27]. Patch pipettes were prepared from 1.5 mm thin-walled borosilicate glass tubes using a Sutter micropipette puller P-97 and had typical resistances of 4–6 MΏ when filled with an internal solution containing (mM): 110 K^+^ aspartate, 20 KCl, 1 MgCl_2_, 0.1 Na-GTP, 5 Mg-ATP, 5 Na_2_-phospocreatine, 1 EGTA, 10 HEPES, pH adjusted to 7.3 with KOH. The external Tyrode's bath solution consisted of (mM): 140 NaCl, 5 KCl, 1 CaCl_2_, 1 MgCl_2_, 10 glucose, 10 HEPES, pH adjusted to 7.4 with NaOH. Voltage- and current-clamp recordings were performed at 37°C within 4 to 7 days after LV-miR-X™ transduction. For action potential (AP) recording, the hESC-CMs were stimulated with 0.1–0.5 nA for 1–5 ms. Human ESC-CMs were categorized into pacemaker, atrial or ventricular phenotypes according to standard AP parameters (summarized as Table S1) as we and others previously described [19], [20]. Numeric details of this current work are given in Table S2. Voltage-clamp recordings of ionic currents were performed using standard electrophysiological and pharmacological protocols for isolating the ionic component of interest (see supplemental information for details). Current- and voltage-clamp recordings were performed on the same cells so that the AP phenotypes and electrophysiological properties could be directly examined for correlation.

### Measurements of cytosolic Ca^2+^

Ca^2+^ transients of hESC-derived CMs were made as we previously reported [24], [28], [29]. In brief, hESC-CMs were incubated with 5 µM Fura-2/AM and 0.2% pluronic F-127 for 30 min at 37°C. Fluorescent signals obtained upon excitation at 340 nm (F_340_) and 380 nm (F_380_) were recorded from cells perfusing with Tyrode's solution containing (mM): 140 NaCl, 5 KCl, 1 CaCl_2_, 1 MgCl_2_, 10 glucose, and 10 HEPES. The F_340_/F_380_ ratio was used to represent cytosolic [Ca^2+^]_i_. To elicit cytoplasmic Ca^2+^ transients, hESC-CMs were electrically pulsed (0.1 to 0.5 Hz). Ca^2+^ transients were recorded and analyzed after a series of depolarization that enabled each transient to fully decay so as to establish a steady-state. Data were analyzed using the Ionwizard software (Version 5, IonOptix) to generate the Ca^2+^ transient parameters reported. Amplitude of Ca^2+^ transient is defined as the ratio of F_340_/F_380_. Maximum upstroke velocity and maximum decay velocity were (F_340_/F_380_)/duration of the steepest slopes of the upward and decay phases, respectively.

### Real-time PCR

**Figure 1:**
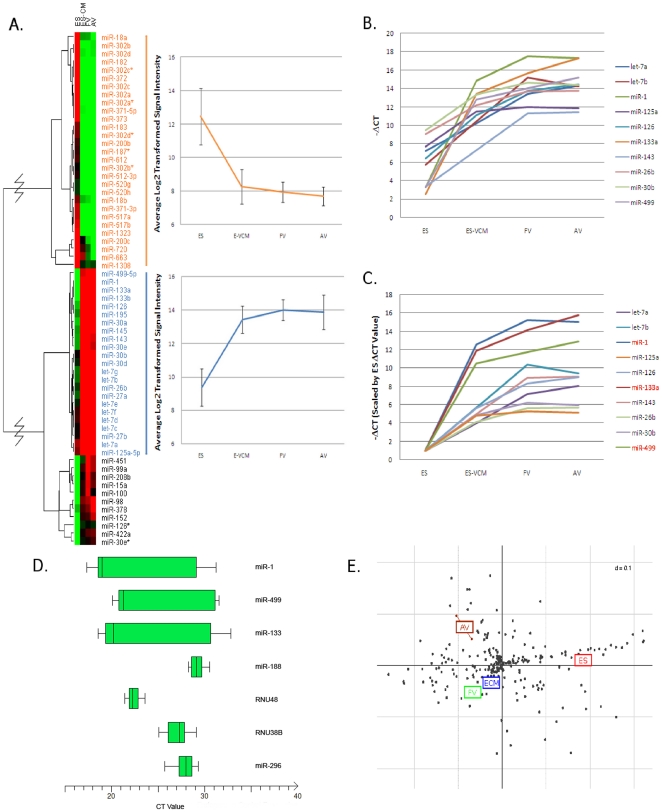
Characterization of miRNAs Present in hESC and Ventricular CMs. (A) A heatmap showing the results of ANOVA differential expression analysis; 63 miRs were differentially expressed between undifferentiated human ESC and hESC-derived VCMs (p<0.05). Red to green indicates high to low expression. Right panels: Average signal intensities of the 29 miRs differentially expressed in hESC (top), and the 23 miRs that displayed a “plateau” pattern across E-, F- and A-VCMs (bottom). (B) “Plateau” miRs validated with quantitative RT-PCR. (C) After normalized with the values of hESCs, miR-1, -133, and -499 displayed the highest differential expression between VCMs and hESCs. (D) MiR-188 and -296 were identified as stably expressed across developmental stages, at levels comparable to standard endogenous controls RNU38B and RNU48. Conversely, the expression of miR-1, -499, and -133 varies greatly across developmental stages. The box plot for each sample summarizes the median, first and third quartiles and range of raw CT values for each miRNA or endogenous control in all cell types assayed. (E) Between-group analysis (BGA) was carried out using principle component analysis (PCA) to provide a visual means to observe the variance of data in specified groups (G) along G-1 number of axes. Data points corresponding to individual miRs are distributed along the axes according to discriminating eigenvectors. Data points and groups that are strongly associated will be clustered within the graph due to their similar projection (both direction and distance) from the origin. Details on the calculations used in BGA can be found in Culhane et al. (2002) [38].

Total RNA was extracted with RNeasy Mini kit (Qiagen) and measured (A260/A280) by spectrophotometry. Reverse transcription was done with the QuantiTect Reverse Transcription Kit (Qiagen). Quantitative PCR was carried out using Platinum SYBR green qPCR SuperMix-UDG (Invitrogen) and MyiQ Optical Module (Bio-Rad) according to the manufacturer's instructions. Primers were designed using Oligo Perfect Software (Invitrogen, Table S3). To validate miR overexpression, reverse transcription was done with the QuantiMiR Reverse Transcription Kit (System Biosciences). Quantitative PCR was carried out using Platinum SYBR green qPCR SuperMix-UDG (Invitrogen) and MyiQ Optical Module (Bio-Rad). The forward primer was using the same sequence of mature miRs, and the universal reversed primer was provided in the QuantiMiR Kit. In triplicate, select miRs were compared against undifferentiated hESCs as well as the 4 endogenous controls, nucleolar RNAs RNU38B and RNU48 (as recommended by the array manufacturer) and the stably expressed miRs, miR-188 and miR-296-5p (as identified in our own experiments) (Figure 1D). Of note, miR-188 was shown to display the most stable expression profile across all cell types examined. For each cell type, the −ΔCT was calculated for each miR (−ΔC_T_ = −1*[median sample C_T_−median control C_T_]) paired with each of the 4 endogenous controls. In other words, four −ΔCT values were individually generated for each miR of the four types investigated. Each −ΔC_T_ value was then scaled by the background −ΔC_T_ value, which had been termed −ΔC_T0_ (where ΔC_T0_ = ΔC_T background_−ΔC_T control_; assuming a C_T_ of 35 or higher is equal to zero expression). Any miR with a C_T_≥35 was rounded to 35. ANOVA analysis was performed and significant differences had p values less than or equal to 2.59E-5.

### MTT assay for cell viability

Cell viability of control or experimental groups was determined using a colorimetric 3-(4, 5-dimethylthiazolyl-2)-2, 5-diphenyltetrazolium bromide (MTT) kit (Roche Diagnostics). In brief, MTT (10 µl) labeling reagent (5 mg/ml in PBS) was added to cells, followed by incubation at 37°C for 4 hours. Solubilization solution (100 µl) was added to dissolve the formazan crystals formed. Absorbance at 540 nm was measured by a spectrophotometer.

### MiR Target Analysis

Predicted mRNA targets of miRs were identified using three algorithms, namely miRanda [30], TargetScan 5.0 [31], and PicTar [32]. Because miRs are negative transcriptional regulators, the list of candidate mRNA targets were further filtered for pathway analysis by a reciprocal relationship between miRs and mRNA transcripts as further elaborated later. To improve the rate of false positive detection at low expression levels, candidates were only analyzed if the transcripts had at least a two-fold difference in expression level between undifferentiated hESC and each of hE-, hF- and hA-VCMs at normalized intensity values of 1000 or greater or at least a three-fold difference in expression level when intensity values are less than 1000.

### Statistics

Data are expressed as mean±S.E.M. Statistical significance of differences in means was estimated by unpaired Student's *t* test, or Chi-square (*x* ^2^) test, or ANOVA analysis. *P*<0.05 was considered statistically significant.

The authors had full access to the data and take responsibility for its integrity. All authors have read and agreed to the manuscript as written.

## Results

### Differentially expressed miRs in hESC-derived, fetal and adult ventricular CMs, but not undifferentiated hESCs

We hypothesized that cardiomyogenic associated miRs would be differentially expressed in differentiating, developing, and terminally mature CMs. As a first step, we chronologically mapped the miR profiles of undifferentiated hESCs, hESC-derived (hE), fetal (hF) and adult (hA) ventricular (V) CMs. Figure 1A shows that 63 miRs were differentially expressed between hESCs and hE-VCMs (p<0.05). Of these, 29 showed signal intensities at least four-fold higher in hESCs than any of hE-, hF- and hA-VCMs (Figure 1A, top right panel). Particularly, the miR-302 cluster (miR-302a, -302a*, -302b, -302b*, -302c, -302c*, -302d, and -302d*) located within intron 8 of LARP7 and the 371/372/373 cluster (miR-371-3p, -371-5p, -372, and -373) located within an intergenic region of chromosome 19 have been previously shown to associate with pluripotency [33]–[35]. The remaining 34 of 63 differentially expressed miRs were all higher in hE-VCMs. Importantly, only twenty-three miRs continued to be highly expressed in hF- and hA-VCMs (Figure 1A, bottom right panel), suggesting a potential role in specification or maturation. Their signal intensities were at least four-fold higher in hE-VCMs compared with hESCs, and remained at the same relative high levels throughout development into hF- and hA-VCMs. Of note, miR-30a, -30b, -30d, -30e are derived from different regions of the genome but have the same seed sequence (i.e. nucleotides 2–7 on the mature miRs) which is crucial for target selection during RNA interference-mediated inhibition or degradation [36], [37]. This holds true for the miR-27, -133, and -let-7 families, with each family sharing their own identical seed sequences. The profiles of miR-1, let-7a, let-7b, miR-26b, miR-30b, miR-125a, miR-126, miR-133a, miR-143, and miR-499 in hE/F/A-VCM were confirmed by qPCR (Figure 1B). MiR-let-7a, -let-7b, -26b, -125a and -143 were also significantly expressed in human fibroblasts (data not shown), indicating their non-cardiac specificity.

The ventricular-restricted expression pattern of miR-1, -30b, -126, -133, and -499 starkly contrasted the reverse pattern of pluripotency-associated miRs that were differentially expressed in hESCs, and the stable expression levels of miR-188 and -296, which remained relatively unchanged across the different developmental stages examined as reflected by the small variances (Figure 1A, D). Indeed, the stability of miR-188 and -296 was comparable to the endogenous controls RNU38B and RNU48. Quantitative PCR confirmed these patterns and further showed that among all the plateau miRs identified (cardiac-specific or not), miR-1, -133, and -499 were *most* differentially expressed in hE-, hF- and hA-VCMs relative to hESCs after scaling the ΔCT values of each VCM type by the corresponding hESC value (with ratios of 15.0, 15.8, and 12.9, respectively versus 5.1 to 9.4 of the other seven miRs; Figure 1C). As anticipated from the data presented above, principal component analysis revealed cell-specific clustering of hESC, hE-, hF- and hA-VCMs (Figure 1E). Between-group analysis (BGA) provides a visual means to observe the variance of the data set. Each sample is grouped along the axes according to similar miR expression. Figure S2 summarizes our criteria for selecting miR-1, -133 and -499 for further experiments.

### PRE-cardiac differentiation alteration of miRs

To test the possibility that miRs expressed in hE-CMs but not hESC and continued to increase in their expression levels in hF- and hA-VCMs are crucial for ventricular specification and maturation, we systematically performed lentivirus (LV)-mediated transduction for over-expressing these miRs individually in hESCs and hE-CMs to shed insights into their roles before and after cardiac induction, respectively. Compared to controls (WT, LV-BLANK), stable transduction of hESCs by LV-miR-499 or -1 did not affect the transcript expression of pluripotency genes (Oct4, Nanog) or upregulate cardiac genes (α myosin heavy chain or MHC, β myosin heavy chain, troponin T) that are not expressed in the undifferentiated state (p>0.05). These results suggested that overexpression of miR-499 or -1 alone was insufficient to drive cardiac differentiation or compromise pluripotency. Upon cardiac differentiation of stably LV-miR-1-transduced hESCs by EB formation, however, all of α-MHC and β-MHC were significantly upregulated (2- and 3-fold increases, respectively) compared to EBs derived from control WT hESCs (p<0.05). This observation was consistent with the upregulation of NKX2.5 seen in EBs differentiated from LV-miR-1-transduced, but not LV-miR-133-transduced or WT, H7 hESCs that Srivastava and colleagues reported [8]. Similar to the pro-cardiogenic role of miR-1, β-MHC also became significantly upregulated (2.5-fold; p<0.05) in EBs from stably LV-miR-499-transduced hESCs, although α-MHC was unaffected (p>0.05). By contrast, the expression levels of α-MHC and β-MHC were not different between WT and LV-anti-miR-499 hESC-derived EBs. Taken collectively, these results suggest that miR-1 and -499 play similar but distinct roles. The experiments that follow were designed to further dissect their similarities and differences.

### POST-cardiac differentiation alteration of miRs

To study the roles of miR-1 and -499 at a later stage of cardiac induction and chamber specification, we next transduced 20-day old cardiospheres derived by directed differentiation of WT hESCs [17]. When normalized to the control group, transduction of hE-CMs by LV-miR-1, -133 and -499 led to significant 27.4±1.4-, 2.5±0.3- and 20.7±2.2-fold increase in the corresponding miRs, respectively (p<0.05). To assess any cytotoxic effect that miR-1, -133 or -499 expression might have on hE-CMs, a colorimetric MTT assay for cellular metabolisms was performed. No significant differences were observed among the control and experimental LV-miR-1-, 133- and -499-transduced groups (p>0.05; Figure S3).

**Figure 2:**
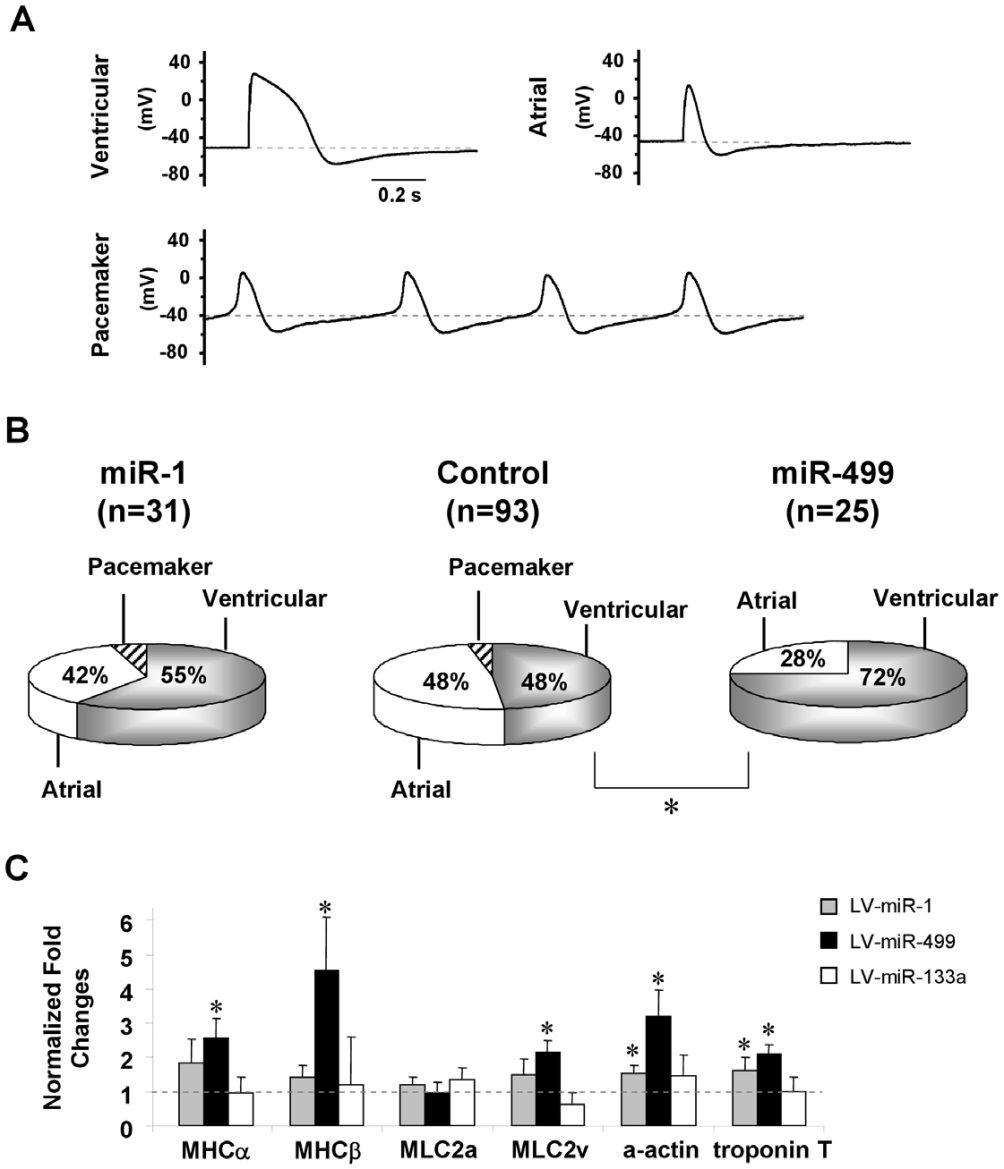
Electrophysiological and Molecular Properties of Control and miR-1, -133, and -499 Transduced hE-VCMs. A) Representative tracings of ventricular, atrial and pacemaker action potentials (APs) of control hESC-CMs. B) The percentage distribution of ventricular, atrial and pacemaker phenotypes before and after LV-miR-1 or -miR-499 transduction. C) Transcriptional expression of cardiac sarcomeric genes in LV-miR-1-, 133- and 499-transduced hESC-CMs. *, p<0.05.

**Figure 3:**
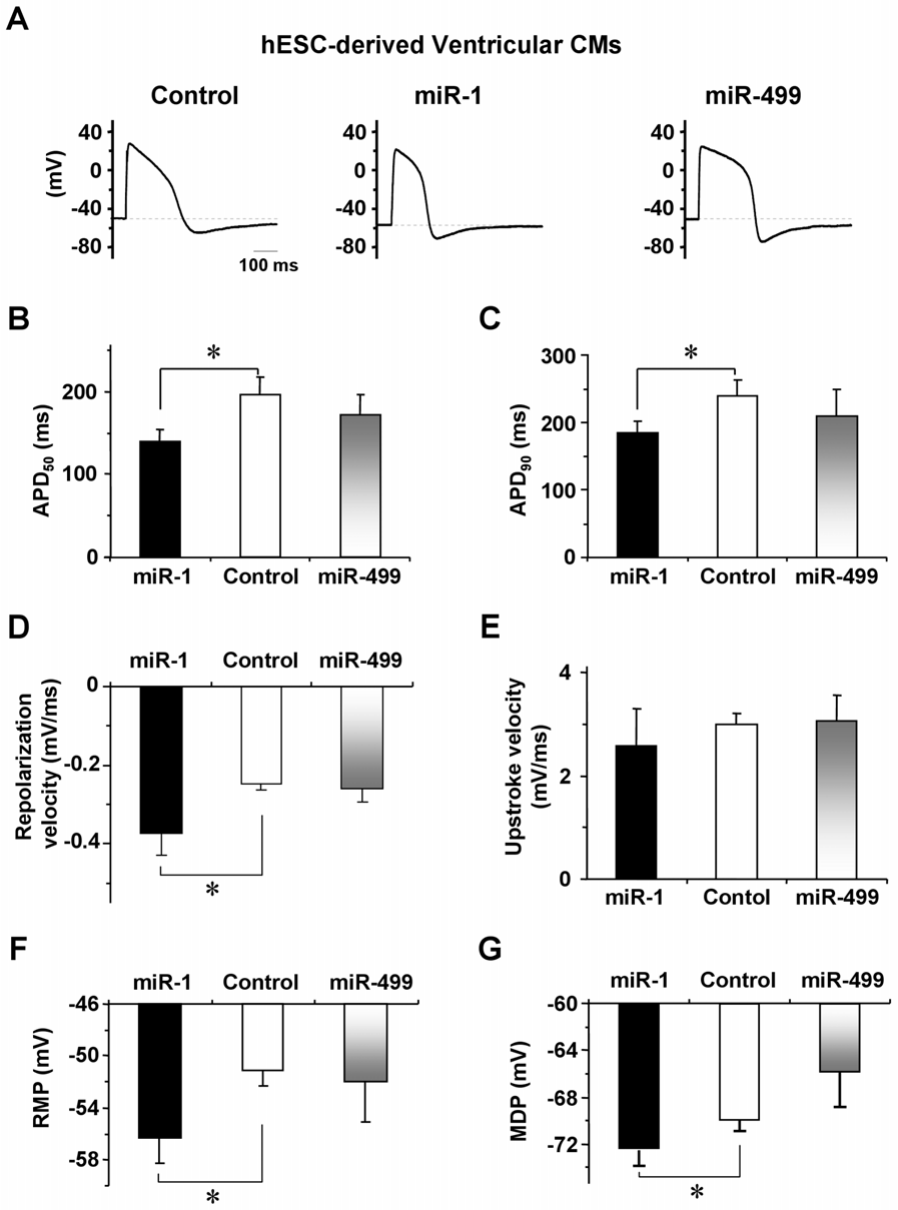
AP Parameters of Control and miR-1 and -499 Transduced hE-VCMs. A) Representative AP tracings of Control, LV-miR-1- and -miR-499-transduced hESC-derived ventricular derivatives as labeled. B-G) Bar graphs summarizing the AP parameters APD_50_, APD_90_, repolarization velocity, upstroke velocity, resting membrane potential (RMP), and maximum diastolic potential (MDP) of the groups in A).

Under control conditions, signature ventricular, atrial or pacemaker APs were observed by patch-clamp recordings of dissociated single hE-CMs (Figure 2A–B; n = 93). The percentage of ventricular AP observed was 48.5% (45 of 93 cells). Consistent with an immature embryonic-like phenotype, APs of hE-VCMs had a depolarized resting membrane potential (RMP, −51.4±1.1 mV, n = 45) or maximum diastolic potential (MDP, −69.7±1.0 mV), with slowed upstroke and decay velocities (3.0±0.2 mV/ms and −0.25±0.02 mV/ms, respectively, n = 45) (Figure 3). Interestingly, LV-miR-499 transduction significantly increased the yield of the ventricular lineage to 72.0% (18 of 25 cells, p<0.05; Figure 2B) without affecting the various AP parameters measured (Figure 3; p>0.05). Consistent with our pre-differentiation transduction experiments already presented, LV-miR-499 transduction of hE-CMs likewise significantly upregulated α-MHC, β-MHC, as well as myosin light chain (MLC) 2v, α-actin and troponin T (p<0.05; Figure 2C). In particular, the increase of β-MHC was the highest (∼5-fold), consistent with a pro-ventricular effect. By contrast, none of miR-1, -133 and –anti-499 exerted any effect on contractile proteins (p>0.05). LV-miR-1 (55.0% or n = 17 of 31, p>0.05), −133 (52.9% or n = 9 of 17, p>0.05) and –anti-499 (44.4% or n = 4 of 9, p>0.05) transduction also did not affect the ventricular yield.

Although the ventricular yield was unchanged by miR-1, however, LV-miR-1 transduction of hE-CMs appeared to uniquely facilitate electrophysiological maturation: APD_50_ and APD_90_ decreased from 197.0±20.7 ms and 240.8±23.1 ms in control hE-VCMs to 139.7±14.2 ms and 174.7±16.6 ms (n = 17, p<0.05; Figure 3B–C) after miR-1 transduction, respectively. The changes were due to a hastened repolarization (p = 0.008; Figure 3D) although the upstroke velocity was not altered (p>0.05; Figure 3E). Furthermore, RMP and MDP became significantly hyperpolarized to −56.6±1.9 mV (p = 0.026) and −73.2±1.3 mV (p = 0.024), respectively (Figure 3F–G). LV-anti-miR-1 did not affect these AP parameters (Figure S5). Neither LV-miR-1 nor -499 had effects on hE-derived atrial CMs when their percent distribution and AP parameters were assessed (Figure 2, Figure S4 and Table S2), suggesting that the effects observed were ventricular-specific. Indeed, miR-21 and -29, robustly expressed in adult CMs (>5-fold higher than hE- and hF-CMs) according to our profiling data confirmed by qPCR, when overexpressed in hESC-CMs by LV transduction, did not alter any of the action potential properties assessed (data not shown). Collectively, our results indicated that miR-1 and -499 had specific and differential effects on ventricular specification and maturation.

### Ionic Basis of the effects of miR-1 on hESC-VCMs

**Figure 4:**
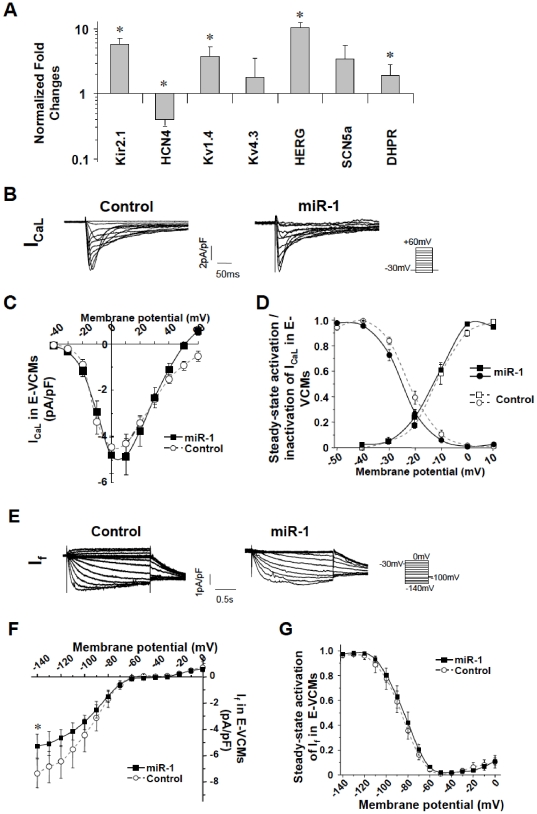
Effects of miR-1 transduction on electrophysiological and molecular properties of hESC-derived CMs. A) Transcriptional expression of sarcolemmal ion channels (Kir2.1, HCN4, Kv1.4, Kv4.3, HERG, SCN5A, DHPR) in hESC-CMs after LV-miR-1 transduction. B) Representative tracings of I_Ca,L_ of control and LV-miR-1-transduced hE-VCMs as labeled. Currents were elicited by the electrophysiological protocol given in the inset. Currents shown were nifedipine-sensitive. C–D) The corresponding current-voltage (I–V) relationship, steady-state activation (circle) and inactivation curves (square) of I_Ca,L_. E) Representative tracings of I_f_ recorded from control and LV-miR-1-transduced hE-VCMs. Currents were elicited by the electrophysiological protocol given in the inset. Currents shown were ZD7288-sensitive. F–G) The corresponding current-voltage (I–V) relationship and steady-state activation curve of I_f_. * p<0.05.

**Figure 5:**
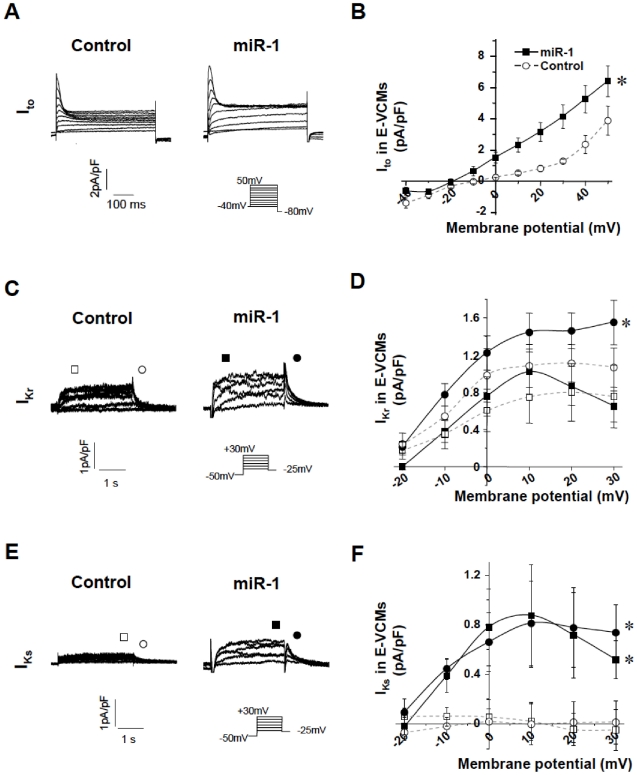
Currents and Current-Voltage Relationships in Control and miR-1 Transduced hE-VCMs. A, C and E) Representative tracings of I_to_, I_Kr_ and I_Ks_ recorded from WT, LV-miR-1-transduced E-VCMs as labeled. Currents were elicited by the electrophysiological protocol given in the inset. Currents shown were 4-Aminopyridine-, Chromanol 293B- and E4031–sensitive, respectively. B, D and F) The corresponding current-voltage (I–V) relationships. *, p<0.05.

Different ionic channels are known to underlie the different phases of ventricular APs. To investigate these potential mechanisms, we sought to identify changes in ionic components after transduction by probing for the transcriptional changes of several ion channels (Kir2.1, HCN4, Kv1.4, Kv4.3, HERG, SCN5A, L-type Ca_v_ or DHPR) known to be important in ventricular biology. Figure 4A shows that LV-miR-1 transduction led to significant (p<0.05) up-regulation of the Kir2.1, Kv1.4, HERG, and DHPR transcripts and down-regulation of HCN4. The Kv4.3 and SCN5A transcripts were unaffected (p>0.05). Since changes at the transcript level do not always translate into functional changes, we performed voltage-clamp recordings. Figure 4B shows that LV-miR-1-transduced hE-VCMs expressed nifedipine-sensitive (5 µM) L-type Ca^2+^ channels (I_Ca, L_), a depolarizing component that underlies the Phase 2 plateau phase, with current densities, steady-state activation and inactivation properties not different from control hE-VCMs (p>0.05). Of note, I_Ca, L_ expressed in hE-VCMs were comparable to those found in hA-VCMs. ZD7288-sensitive (30 µM) hyperpolarization-activated pacemaker current (I_f_) that is crucial for Phase 4 depolarization [26], [39]–[42] not seen in normal healthy hA-VCMs but present in hE-VCMs, was modestly yet significantly reduced (p<0.05, n = 7; Figure 4E–F) consistent with HCN4 transcript down-regulation. Steady-state activation curve was unaltered (p>0/05; Figure 4G). No I_K1_ could be recorded (n = 6). Na_v_ currents were also not different (data not shown). Hyperpolarizing K^+^ currents that are crucial for repolarization such as the transient outward current (I_to_), the slow (I_Ks_) and rapid (I_Kr_) components of the delayed rectifier, pharmacologically separated by their specific blockers 4-aminopyridine (4AP; 100 µM), Chromanol 293B (30 µM) and E4031 (10 µM), respectively, were weakly expressed or absent in control hE-VCMs but became significantly augmented after LV-miR-1 transduction (Figure 5, p<0.05).

### MiR-1 improves Ca^2+^-handling properties

**Figure 6:**
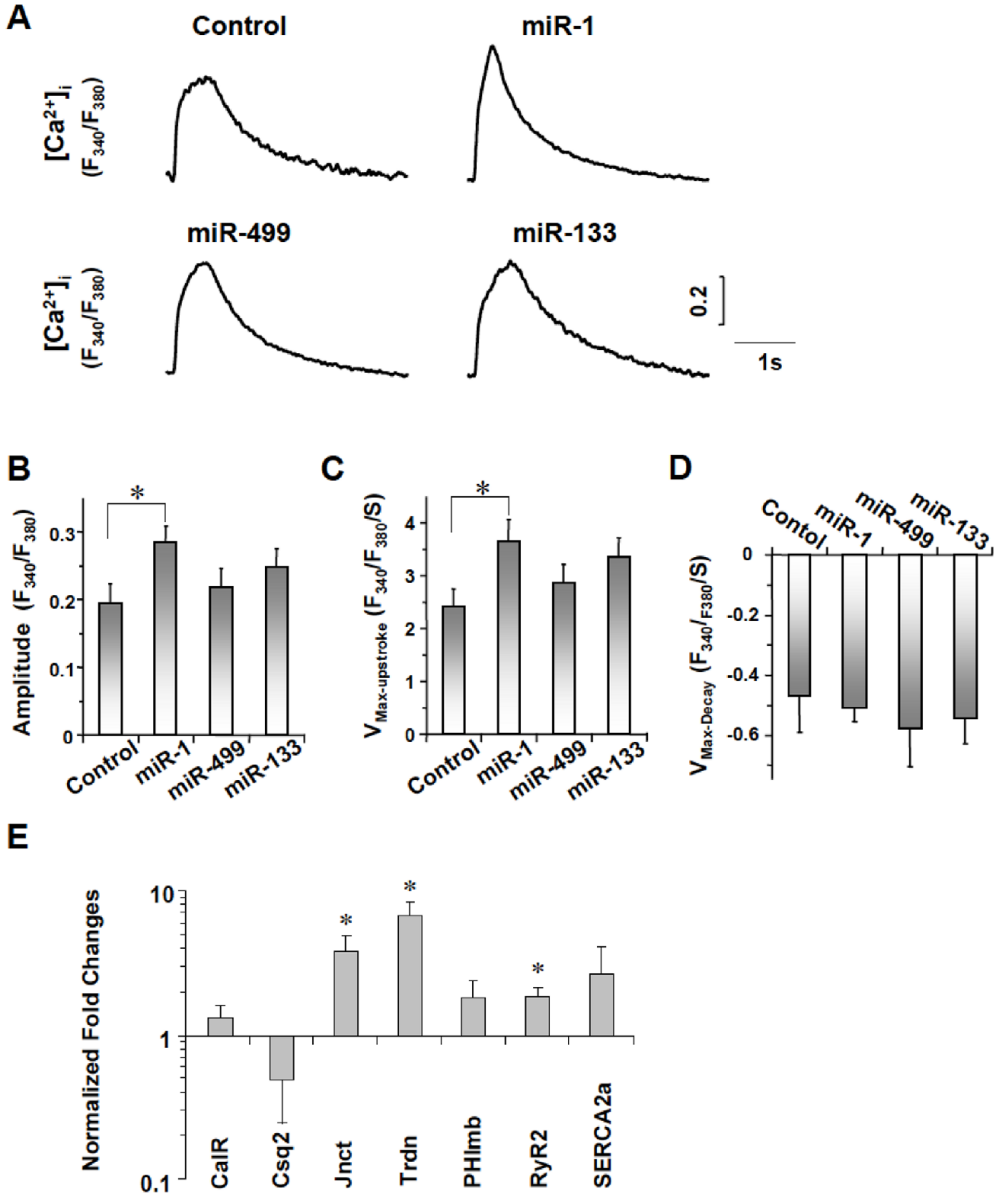
Calcium Handling in Control and miR-1, -133, and -499 Transduced hE-VCMs. A) Representative tracings of Ca^2+^ transients recorded from control, LV-miR-1-, 133- and 499-transduced hESC-CMs. B–D) Comparison of amplitude (B), maximum upstroke velocity (V_max-upstroke_, C), and maximum decay velocity (V_max-decay_, D) of Ca^2+^ transients in control and LV-miR-transduced hESC-CMs as indicated. E) Transcriptional expression of Ca^2+^-handling proteins. *, p<0.05.

Previous reports have indicated that hE-CMs display immature Ca^2+^-handling properties with smaller Ca^2+^ transient amplitudes and slower kinetics (rise and decay) than those of the adult counterparts [24], [28], [29]. Compared to control hE-CMs (n = 26), LV-miR-1 (n = 17) but not −133 (n = 18), −499 (n = 26), anti-1 (n = 12), or -anti-499 (n = 15) transduction significantly augmented the transient amplitude and hastened the upstroke velocity (Figure 6A–D and S6), rendering these parameters more adult-like. However, the decay velocity was unaltered (p>0.05). Consistently, after LV-miR-1 transduction, the transcripts of junctin (Jnct), triadin (Trdn) and ryanodine (RyR2) that play a role in Ca^2+^ release were significantly up-regulated (p<0.05) whereas that of SERCA2a for Ca^2+^ re-uptake was not affected (p>0.05; Figure 6E). Calreticulin (CalR), calsequestrin 2 (CSQ2) and phospholamban (Phlmb) were also unaltered (p>0.05).

### Target and pathway analyses of MiR-1 and -499

Next we performed target and pathway analyses of miR-1 and -499. Using established algorithms, we generated a list of 1448 and 1226 predicted mRNA targets for miR-1 and -499, respectively. These predicted target genes were analyzed for relevant Gene Ontology (GO) classifications and Map Annotation and Pathway Profile (MAPP) pathways involved in CM functions such as hypertrophy, ion channel regulation, contraction, etc. The numbers of genes in each of these select pathways and the numbers of predicted miR-1 and -499 targets in these pathways are given in Table S4. Indeed, miR-499 and miR-1 shared a number of overlapping targets including those that are known to play important roles in early cardiogenesis. Among these, GATA4 is a predicted target of miR-499 but not miR-1. Consistent with this prediction, transduction of hE-CMs by LV-miR-499, but not LV-anti-miR-499, led to a 3-fold downregulation of GATA4 (p<0.05). Interestingly, LV-miR-1-transduction of hE-CMs significantly upregulated GATA4 (by 15-fold; p<0.05). In stark contrast, MESP1 was affected by none of LV-miR-1, miR-499 or –anti-miR-499 (p>0.05). Interestingly, LV-miR-499, but not -anti-499 or –miR1, upregulated MEF2C (by ∼2.5-fold, p<0.05).

**Figure 7:**
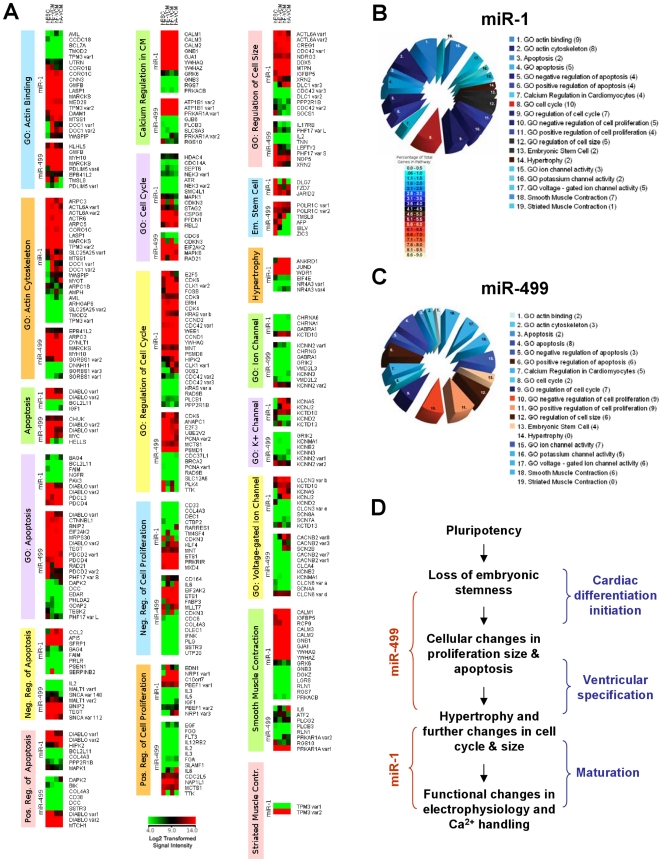
Analysis of Putative Transcriptomic Effects of miR-1 and -499 Expression in CM Development and Maturation. A) Transcriptomic analysis of predicted targets for miR-1 and -499 involved in select pathways as indicated. All genes were log_2_ transformed and normalized. Cluster analysis was performed with Cluster 3.0 using centroid linkage. Pie charts for B) miR-1 and C) -499 summarizing the number of genes (in parentheses) in each selected pathway or Gene Ontology (GO) classification that were found to agree with our transcriptomic data. See text for further details. The color gradient corresponds to a percentage (% = number of genes in each group meeting criteria/total number of genes in pathway or GO classification * 100). D) Summary of the proposed sequence of biological processes that occur during human cardiogenesis and the actions of miR-1 and -499.

To obtain a global view for additional insights, we next performed microarray transcriptomic analysis. Since an abundantly expressed miR is anticipated to down-regulate its target transcripts, we aimed to *experimentally* fine-tune our list of predicted targets by profiling their expression. Figure 7A shows the transcriptional profile heatmaps of miR-1 and -499 predicted targets identified in the selected GO/pathways as indicated. Similar to the reciprocal relationship described for normal and failing adult human CMs [14], we identified multiple functional groups of transcripts that were expressed at low levels (i.e. green) in the miR-1 and miR-499 abundant hE-, hF- and hA-VCMs. The numbers of these genes and their percentages relative to the total genes in each selected pathway or GO classification are summarized in the pie charts given in Figure 7B–C and in Table S4. These putative miR-1 and -499 targets agreed with our transcriptomic data and met one of two criteria: 1) The gene was expressed below a normalized log_2_ value of 1.0 (∼500 signal intensity units) in all CM types assayed *or* 2) The gene was expressed below a normalized log_2_ value of 2.0 (∼1000 signal intensity units) *AND* was expressed at least 2-fold lower in hE-, hF- and hA-VCMs compared with undifferentiated hESCs. According to these analyses, miR-499 is most closely associated with the regulation of embryonic stemness, cell proliferation, cell size and apoptosis; whereas, miR-1 is implicated in control of embryonic stemness, cell cycle, hypertrophy and cell size.

## Discussion

Although miRs have been implicated in heart development, their roles in human heart cells require further studies. In particular, the functional effects of miRs on the electrophysiological and Ca^2+^-handling properties of human CMs have not been investigated. In the present study, we have identified miR-499 and -1 as determinants of ventricular specification and maturation of human CMs, respectively. Consistent with the anticipated multi-faceted roles of miRs, miR-1 and -499 exert multiple but specific effects on a range of ion channel, Ca^2+^-handling and contractile proteins known to be important in ventricular biology. Further analyses suggest that these events were orchestrated consequences of numerous cellular changes directly regulated by miR-499 and -1.

Although miR-499 is up-regulated in human mesenchymal stem cells upon *in vitro* propagation [23], its role and involvement in human cardiogenesis or cardiac differentiation of hESCs has not been demonstrated. Interestingly, miR-499 is located within intron 20 of MYH7B on chromosome 20 and is highly conserved in vertebrates. Our present study reports that miR-499 promotes ventricular specification and significantly augments β-MHC expression in hE-CMs, although it has no effect on the electrophysiological and Ca^2+^-handling properties. By contrast, miR-1 has no effect on the percent yield of VCMs from hESC differentiation, but it uniquely facilitates electrophysiological and Ca^2+^-handling maturation by altering the expression levels of several immature related components (I_to_, I_Kr_, I_Kr_ and I_f_) to levels closer to those of adults. The effect on Ca^2+^-handling was consistent with a recent report of miR-1 enhancing excitation-contraction coupling by increasing phosphorylation of L- type and RyR2 channels [43]. Of note, however, a hyperpolarization overshoot followed by a Phase 4-like depolarization, pro-arrhythmic traits not observed in mature adult VCMs, were present in both control and LV-miR-1-transduced cells, indicating that the pro-maturation effect of miR-1 was at best partial.

In adult rat ventricular myocytes, miR-1 overexpression has been shown to markedly increase I_Ca,L_ [43] and suppress the α and β subunits of I_Ks_ (i.e. KCNQ1 and KCNE1) [44]. However, our experiments showed that hE-VCMs already expressed I_Ca,L_ at a level comparable to that in adult and I_Ca,L_ remained unaltered after LV-miR-1 transduction. By contrast, I_Ks_ was not expressed in hE-VCMs but was up-regulated by LV-miR-1. These results suggest that the effects of miR-1 on VCMs are context-dependent. Furthermore, miR-1 overexpression in hESC-VCMs led to the upregulation of some ionic currents. It is possible that miR-1 targets a transcriptional or translational repressor or negative functional inhibitor of these ion channels, Adding another level of complexity is our previously reported observation that the transcript levels and functionality of ion channels do not necessarily correlated [27] because ion channel functions (gating and permeation) often depend on the presence of accessory units (such as beta subunit) and other factors (e.g., post-translational modification such as glycosylation). Further investigations are required to better understand the underlying relationships.

The two variants of miR-1 identified to date, miR-1-1 and 1-2, share the identical mature sequence and are located within intron 2 of an uncharacterized gene C20ORF166 and intron 12 of the E3 ubiquitin-protein ligase MIB1 (Mindbomb Homolog 1) on chromosomes 20 and 18, respectively. Interestingly, miR-133a has two sequences located within the same introns where miR1-1 and -1-2 are found [45]. As such, miR-1 and -133 are considered single “miR genes” which are transcribed and processed to generate the corresponding mature miRs. Indeed, miR-133 has been implicated in early cardiac differentiation of murine and human ESCs by repressing the non-mesoderm lineages, rather than by directly promoting cardiogenesis *per se* [8]. Consistently, miR-133 exerts no effects on Ca^2+^-handling and contractile proteins when cardiovascular progenitors of later stages were transduced. Taken collectively, the results suggest that key cardiogenic miRs and gene products cluster together at specific chromosomal locations for effective regulations.

Our finding that pre- and post-differentiation transduction with LV-miR-499 similarly led to β-MHC upregulation hints at the possibility that *miR-499 exerts its pro-cardiogenic action after cardiac differentiation is initiated*. This is consistent with the finding that stably LV-anti-miR-499-transduced hESCs were able to efficiently differentiate into hE-CMs with levels of GATA4, α-MHC and β-MHC not different from those of WT EBs. Indeed, MEF2C that is required for contractile protein activation also remains unchanged even after successful stable suppression of miR-499 by LV-anti-miR-499. During the course of our manuscript preparation, van Rooij *et al* reported that a family of miRs encoded by myosin genes, including miR-499, governs myosin expression and muscle performance [46]. Using an elegant transgenic mouse model, they demonstrated that miR-208a is required for expression of β-MHC/miR-499 but its cardiac functions can be replaced by miR-499, suggesting the latter as a downstream mediator. Consistently, Sluijter *et al*. independently identified the upregulation of miR-1 and -499 in beating cardiomyocytes differentiated from human fetal cardiac progenitors [47]. Transient transfection to overexpress miR-1 or -499 in these human progenitors reduces their proliferation by repressing histone deacetylase 4 or Sox6 although no functional properties were reported. Most recently, Hosoda et al shows that miR-499 targets Sox6 and Rod1, traverses gap junction channels and translocates to structurally coupled human cardiac stem cells, enhancing their cardiomyogenesis *in vitro* and after infarction *in vivo* [48]. In another separate study [49], we reported that miR-499 are strongly associated with cardiac differentiation and share many predicted targets with miR-208 that has been previously shown to associate with cardiac development. Furthermore, overexpression of miR-499 or -1 results in upregulation of important cardiac myosin heavy-chain genes in embryoid bodies; miR-499 overexpression also causes upregulation of the cardiac transcription factor MEF2C. Taken collectively, these independent studies and ours strongly suggest an important biological role of miR-499 in cardiac differentiation. Our present study focuses on and further demonstrates the functional implications of miR-499 and -1 in the context of cellular electrophysiological and Ca^2+^-handling properties. We also showed that GATA4 is a probable target of miR-499 but not miR-1. However, our data did not allow us to exclude the possibility that GATA4 down-regulation was merely an indirect or secondary effect. Identification of additional common and distinct targets of miR-499 and -1 requires extensive validation but could help dissect their overlapping and differential roles in cardiac differentiation.

Cardiac differentiation involves at least two processes: 1) downregulation of stemness genes that leads to the loss of pluripotency, and 2) up-regulation of cardiac gene products (such as heart-specific transcription factors, etc) that subsequently drives cardiac differentiation, followed by chamber-specific specification of cardiac progenitors. While the loss of pluripotency is universally required for differentiation into any lineages, cardiac differentiation and specification likely involves multiple heart-restricted factors and miRs. We are proposing that upon the initiation of cardiac differentiation, miR-499 serves to promote ventricular specification. This notion is supported by the finding that miR-499 over-expression increases the ventricular yield, although miR-499 per se may not be absolutely necessary for initiating cardiac differentiation. Indeed, the lack of effect of anti-miR-499 is consistent with its lack of expression in undifferentiated hESCs but a cardiac fate has been acquired.

One possible limitation of the present work is that these our experiments were primarily performed *in vitro* with hESC as our model. In developing organisms, cell fates are influenced by signals from surrounding tissues and in heart, by well-characterized differentiation factors such as Nkx2.5, Hand2, Mef2 and Tbx5 [50]–[53]. In this regard, Langendorff isolated hE-, hF- and hA-VCMs, and *in vitro* hESC- and directed cardiac differentiation-based models, may not completely recapitulate the *in vivo* setting. Indeed, although stably LV-anti-miR-499-transduced hESCs could differentiate into hE-CMs, our preliminary experiments of injecting zebrafish embryos with miR-499 or -1 anti-sense probes, but not blank or scrambled sequences, led to significant anatomical and functional heart defects, suggestive of an *in vivo* role.

An inhibitory effect of miR-499 on atrial specification would also lead to an increased percentage yield of ventricular derivatives and cannot be ruled out, although miR overexpression led to neither cytoxicity nor functional changes of atrial CMs. Furthermore, up-regulated gene expression and/or hypertrophy (without hyperplasia) can lead to some of the results presented (e.g., upregulation of MHC). While our data did not allow us to distinguish between the possibilities, such would still support a pro-ventricular effect.

Of note, we have not examined in this study other non-cardiac-specific miRs such as the let-7 family that have also been identified to display the same differential plateau expression pattern (e.g., in fibroblasts) for their roles in terminal differentiation. It is possible that these miRs play a more generalized role in maturation, which in turn could lead to similar functional changes. Finally, no single algorithm can make miR target predictions with 100% accuracy. Indeed, some functionally defined targets (e.g., IRX5 [5], DLL1 [8], KCNE1 and IKNQ1 [44]) are not predicted by any of the existing algorithms, suggesting that our target list is at best partial.

Based on our collective experimental and bioinformatic data sets, we propose a sequence of biological events that occur during human cardiac differentiation. This is schematically summarized in Figure 7D. The first step requires the loss of pluripotency and embryonic stemness that are mediated by miRs including the miR-302 and -371/372/373 clusters. As cardiac differentiation continues, ventricular specification occurs following cellular changes in cell proliferation, size and apoptosis that are mediated by miR-499. Subsequently, miR-1 regulates physiological hypertrophy and other changes in cell cycle and size, which in turn lead to a series of well-orchestrated functional changes in electrophysiological, Ca^2+^-handling and contractile properties for maturation.

Using hESCs as an experimental model, we have functionally demonstrated that miRs play crucial and specific roles in human cardiac differentiation. MiRs and genes that are crucial to cardiac differentiation and maturation appear to cluster at specific chromosomal locations. The effects of miRs on VCMs dynamically depend on the developmental stages and are context-dependent. We conclude that miR-1 and -499 play differential roles in human cardiac differentiation: While miR-499 promotes ventricular specification in the context of hESC-derived cardiovascular progenitors, miR-1 serves to facilitate their electrophysiological maturation.

## Supporting Information

Figure S1 **After LV-MLC2v-mCherry transduction, only human fetal left ventricular (FLV) CMs but not HEK293 expressed mCherry fluorescence. Bars represent 50 µm.**
(TIF)

Figure S2 **The criteria to select miR-1, -133 and -499 for further experiments.**
(TIF)

Figure S3 **Cell viability of control and transduced hE-CMs assessed by a colorimetric MTT assay.** No significant differences were observed (p>0.05).
(TIF)

Figure S4 Representative AP tracings of Control, LV-miR-1- and -miR-499-transduced hE-ACMs, and bar graphs summarizing the AP parameters of the groups. * p<0.05.
(TIF)

Figure S5 Representative AP tracings of Control (n = 7) and LV-anti-miR-1-transduced (n = 8) hE-VCMs, and bar graphs summarizing the AP parameters of the groups. * p<0.05.
(TIF)

Figure S6 **Representative tracings of Ca^2+^ transients recorded from control (n = 6) and LV-anti-miR-1 transduced hESC-CMs (n = 12), and comparison of the amplitude, maximum upstroke velocity (V_max-upstroke_, U) and maximum decay velocity (V_max-decay_, D) of electrically-induced Ca^2+^ transients of the two groups as indicated.**
(TIF)

Table S1 **AP parameters for classification of CMs subtypes.**
(TIFF)

Table S2 **Human ESC-derived cardiomyocytes (hESC-CMs) action potential properties.**
(TIFF)

Table S3 **The primers were used to quantitatively analysis gene expression.**
(TIFF)

Table S4 **Summary of the numbers of the corresponding predicted targets of miR-1 or -499 in each of these select pathways.** The bracketed numbers indicate the number of genes which expressed at low levels in hE-, hF- and hA-VCMs.
(TIFF)
