# Supplementary figures and images for: Distinct Roles of MicroRNA-1 and -499 in Ventricular Specification and Functional Maturation of Human Embryonic Stem Cell-Derived Cardiomyocytes
Source: PLoS One. 2011 Nov 16;6(11):e27417. doi: 10.1371/journal.pone.0027417 (PMC3217986; doi:10.1371/journal.pone.0027417)

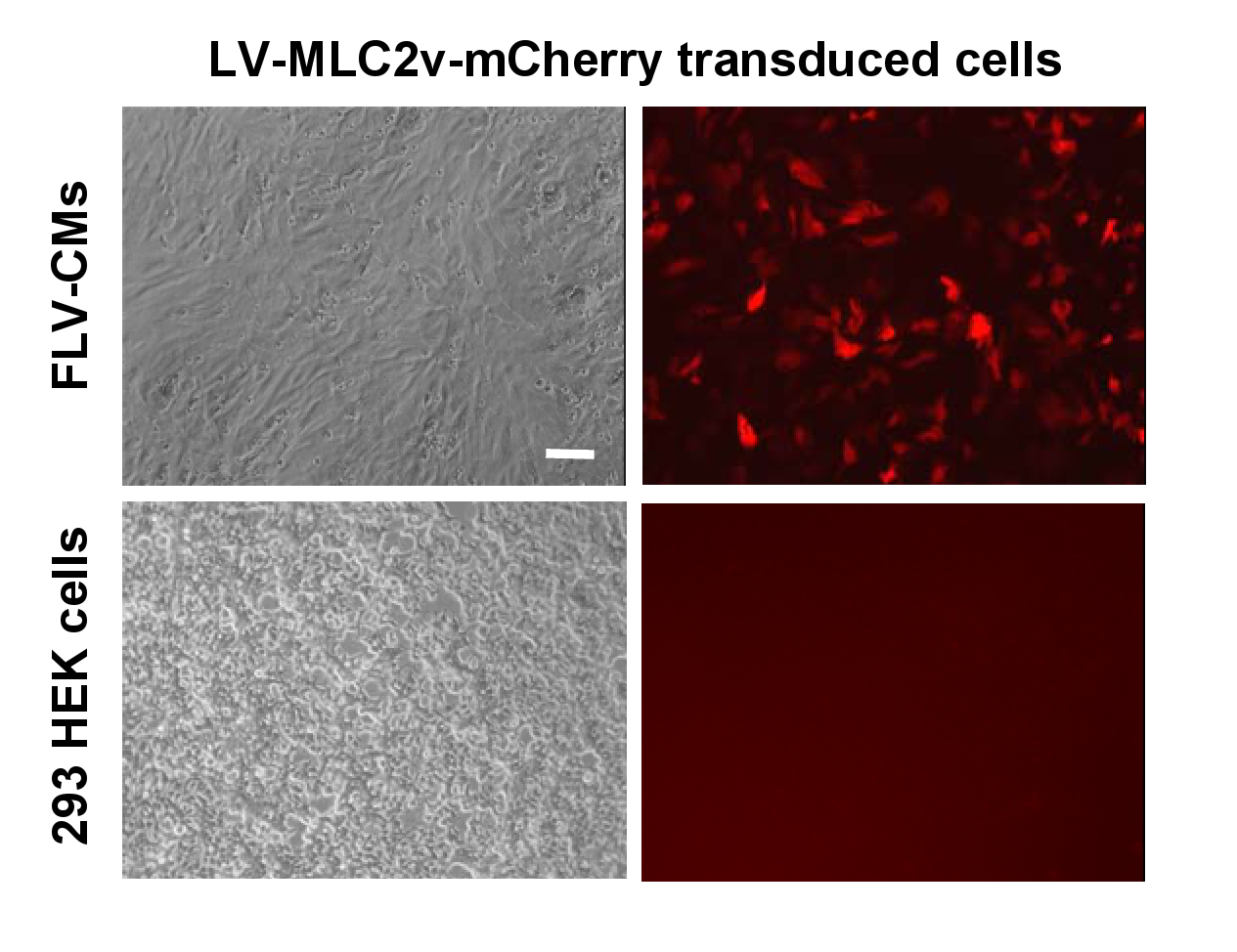

Supplement: Figure S1 — After LV-MLC2v-mCherry transduction, only human fetal left ventricular (FLV) CMs but not HEK293 expressed mCherry fluorescence. Bars represent 50 µm. (TIF) [file pone.0027417.s001.tif]

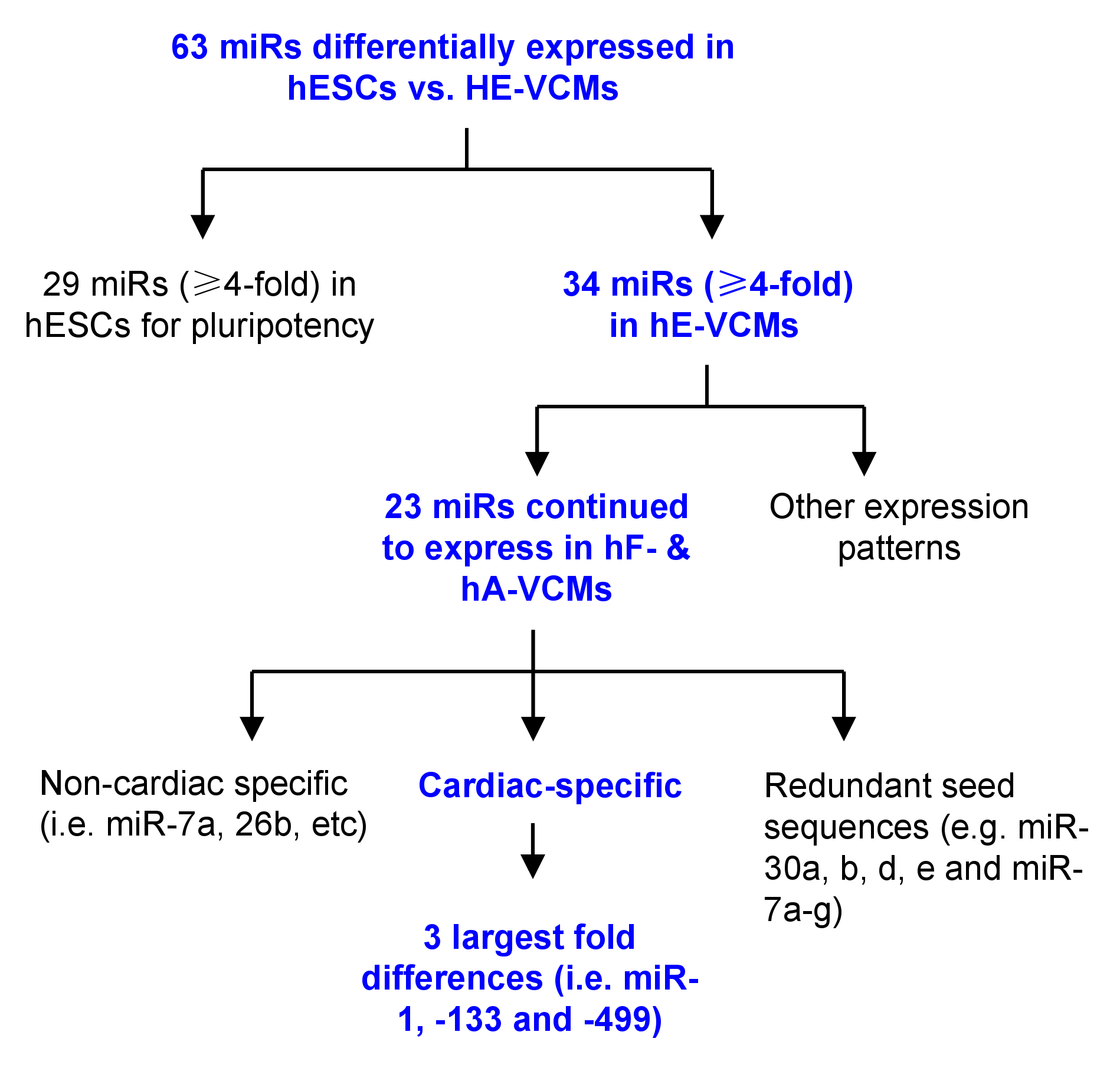

Supplement: Figure S2 — The criteria to select miR-1, -133 and -499 for further experiments. (TIF) [file pone.0027417.s002.tif]

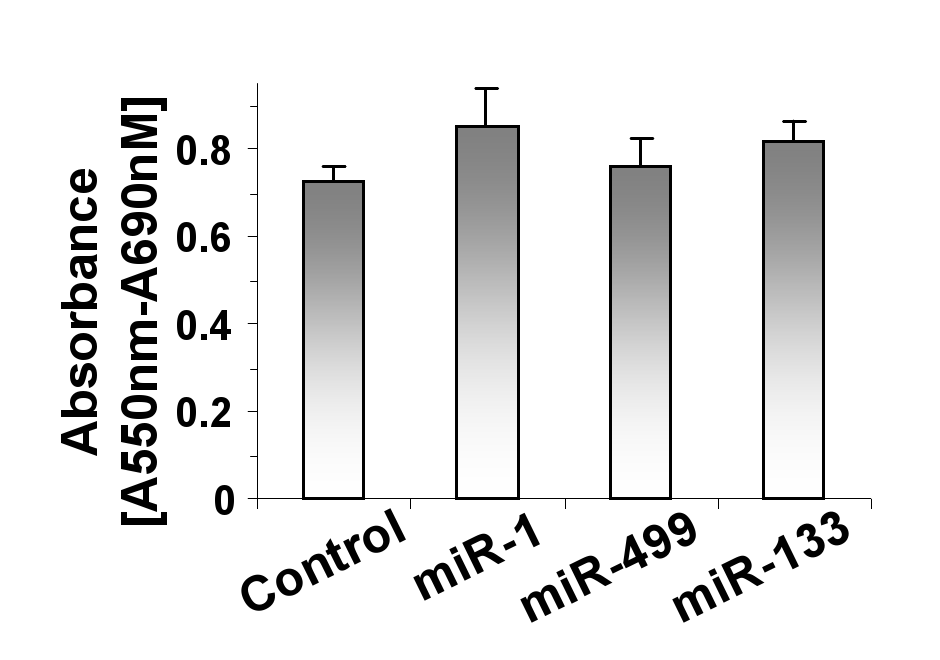

Supplement: Figure S3 — Cell viability of control and transduced hE-CMs assessed by a colorimetric MTT assay. No significant differences were observed (p>0.05). (TIF) [file pone.0027417.s003.tif]

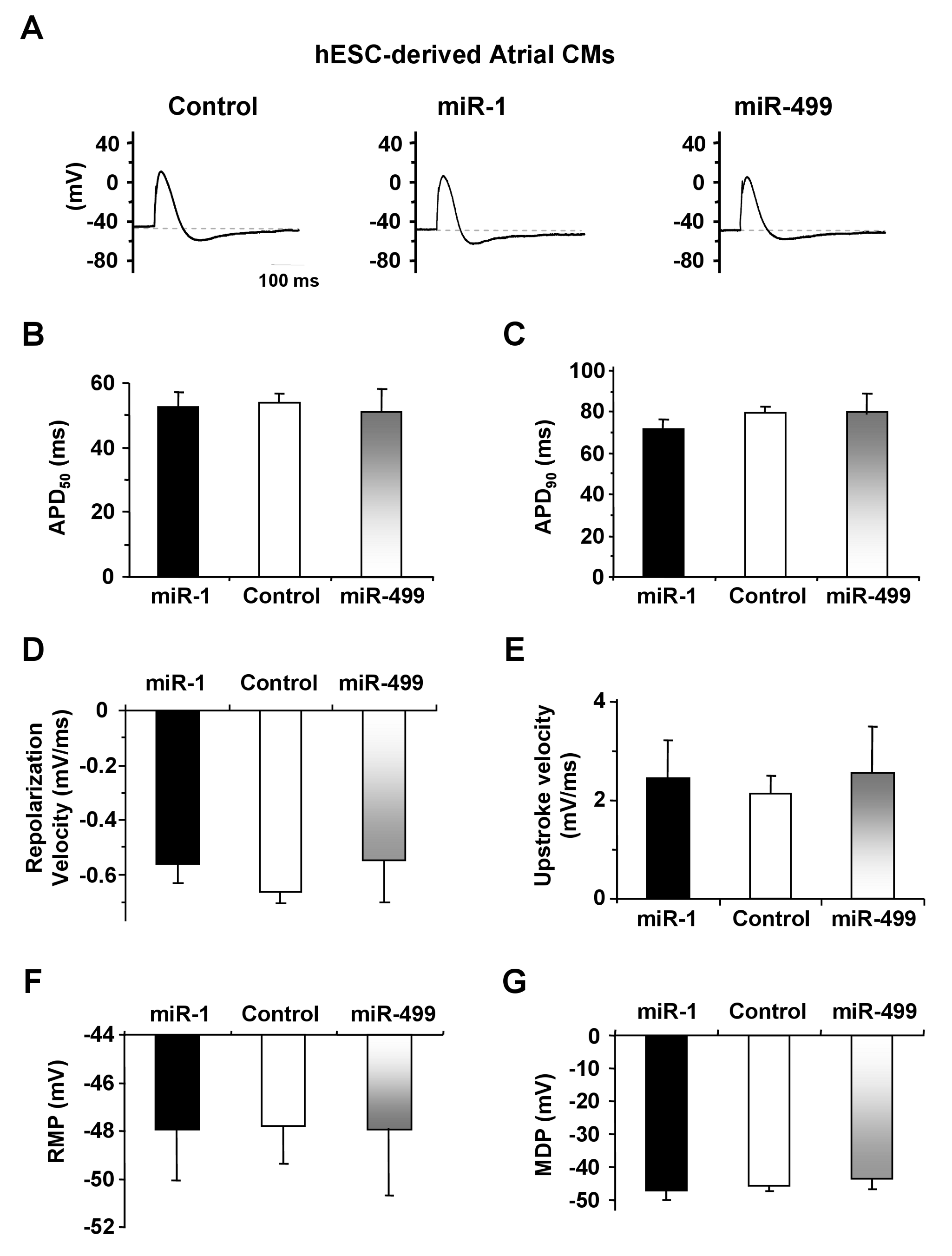

Supplement: Figure S4 — Representative AP tracings of Control, LV-miR-1- and -miR-499-transduced hE-ACMs, and bar graphs summarizing the AP parameters of the groups. * p<0.05. (TIF) [file pone.0027417.s004.tif]

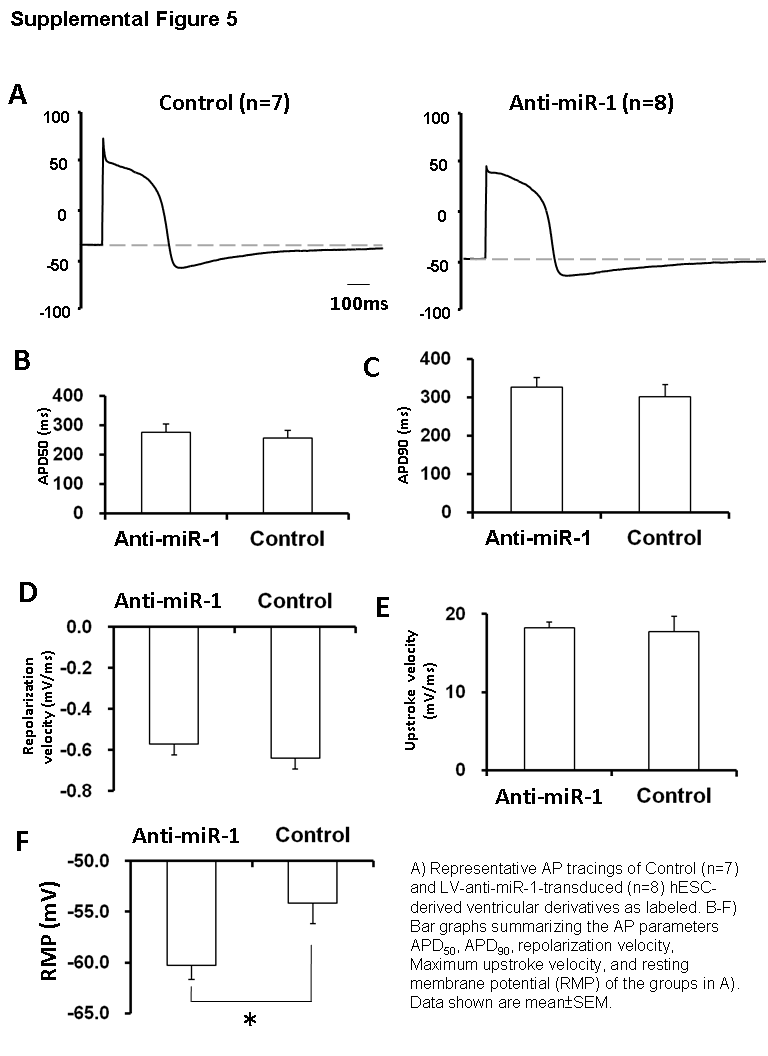

Supplement: Figure S5 — Representative AP tracings of Control (n = 7) and LV-anti-miR-1-transduced (n = 8) hE-VCMs, and bar graphs summarizing the AP parameters of the groups. * p<0.05. (TIF) [file pone.0027417.s005.tif]

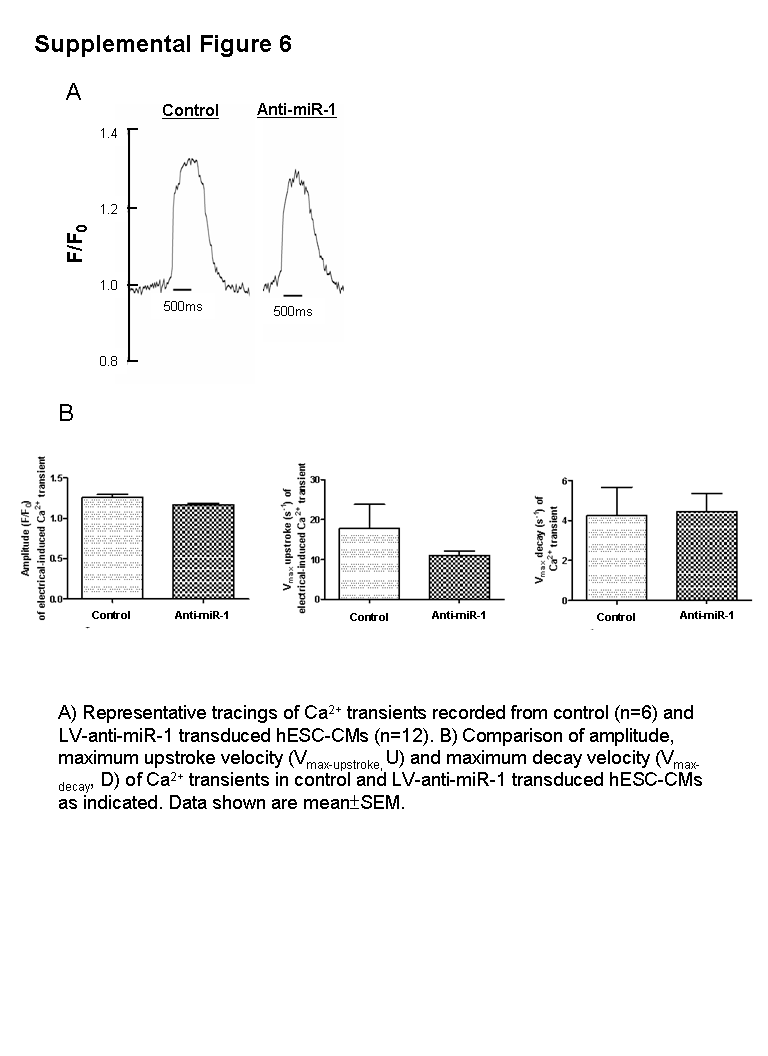

Supplement: Figure S6 — Representative tracings of Ca2+ transients recorded from control (n = 6) and LV-anti-miR-1 transduced hESC-CMs (n = 12), and comparison of the amplitude, maximum upstroke velocity (Vmax-upstroke, U) and maximum decay velocity (Vmax-decay, D) of electrically-induced Ca2+ transients of the two groups as indicated. (TIF) [file pone.0027417.s006.tif]

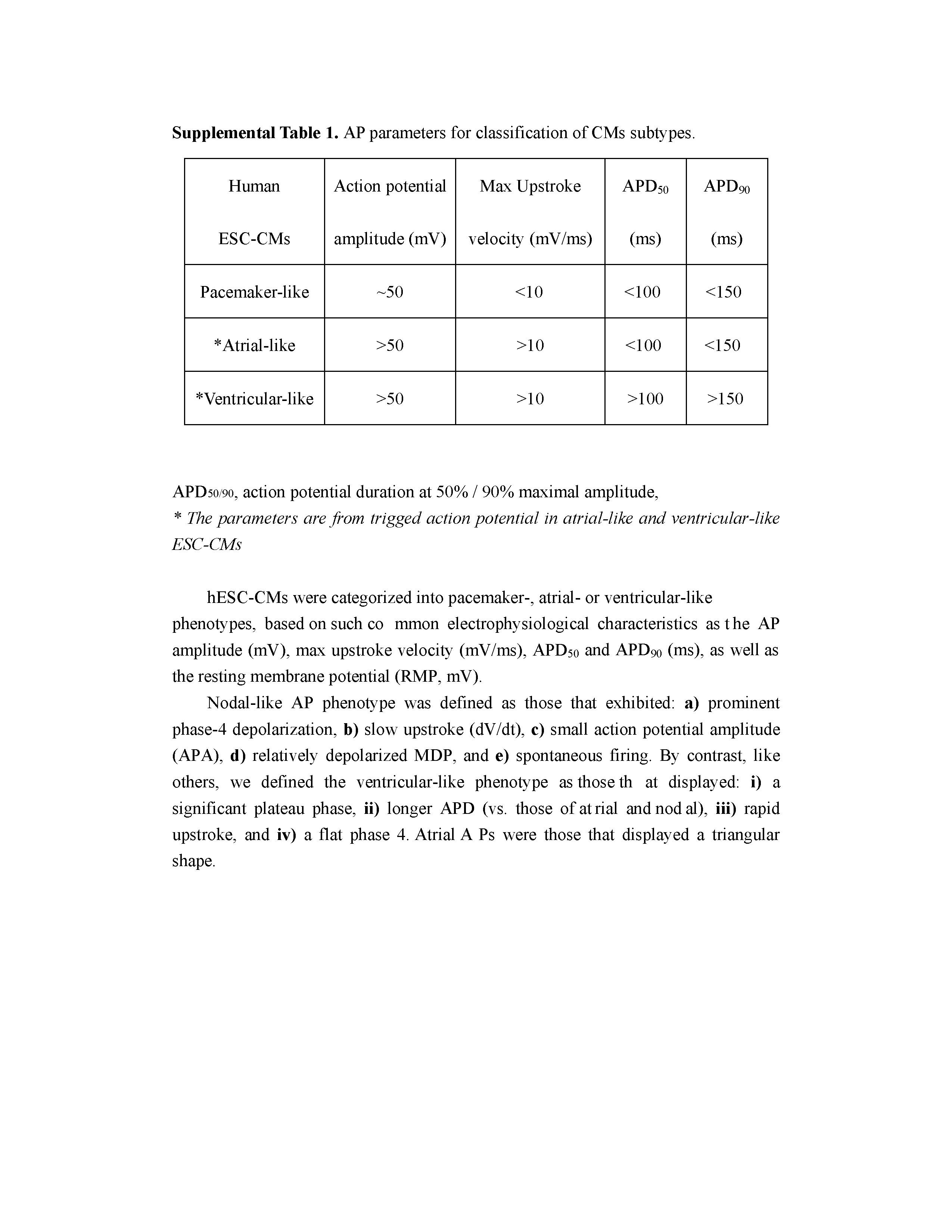

Supplement: Table S1 — AP parameters for classification of CMs subtypes. (TIFF) [file pone.0027417.s007.tif]

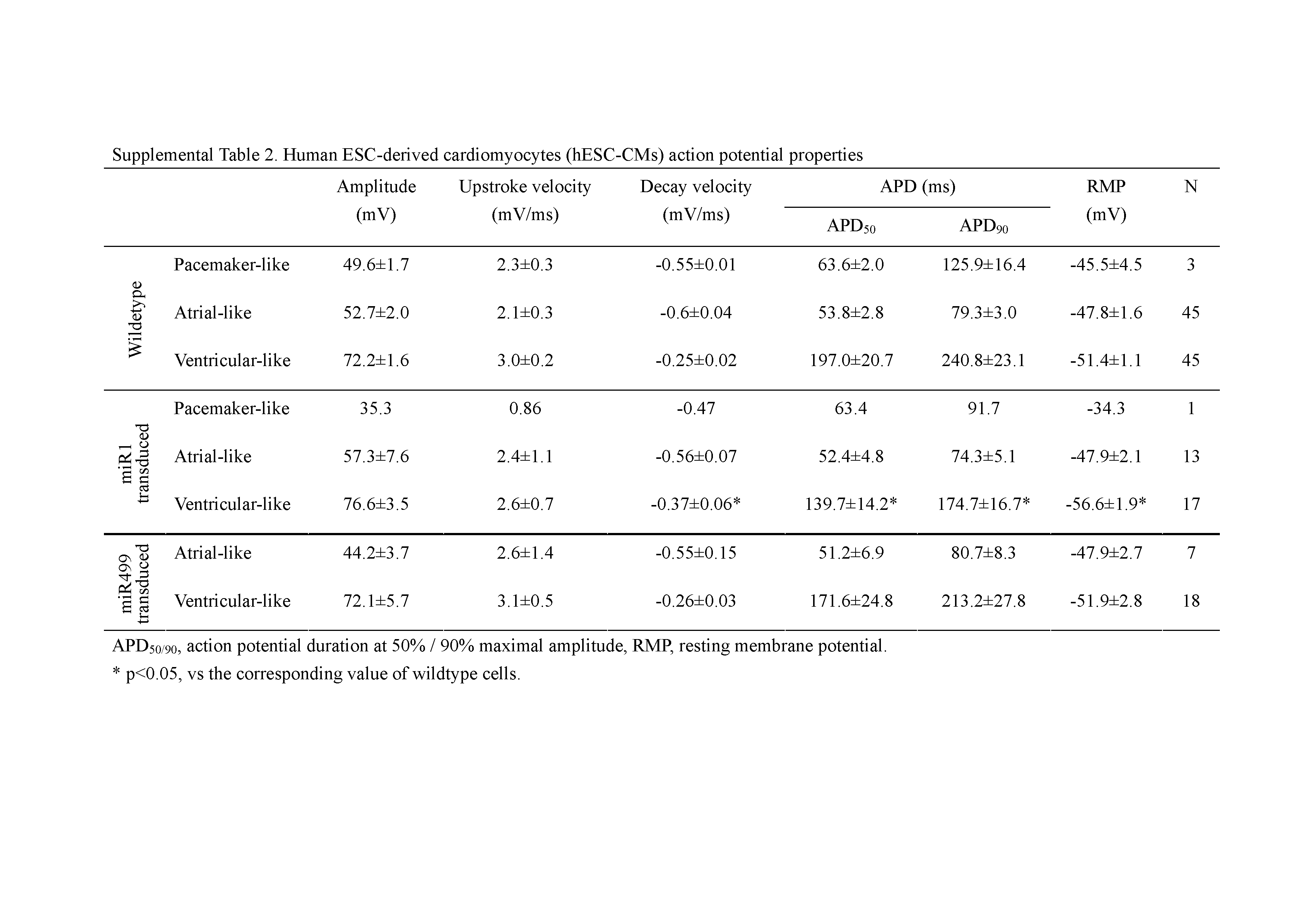

Supplement: Table S2 — Human ESC-derived cardiomyocytes (hESC-CMs) action potential properties. (TIFF) [file pone.0027417.s008.tif]

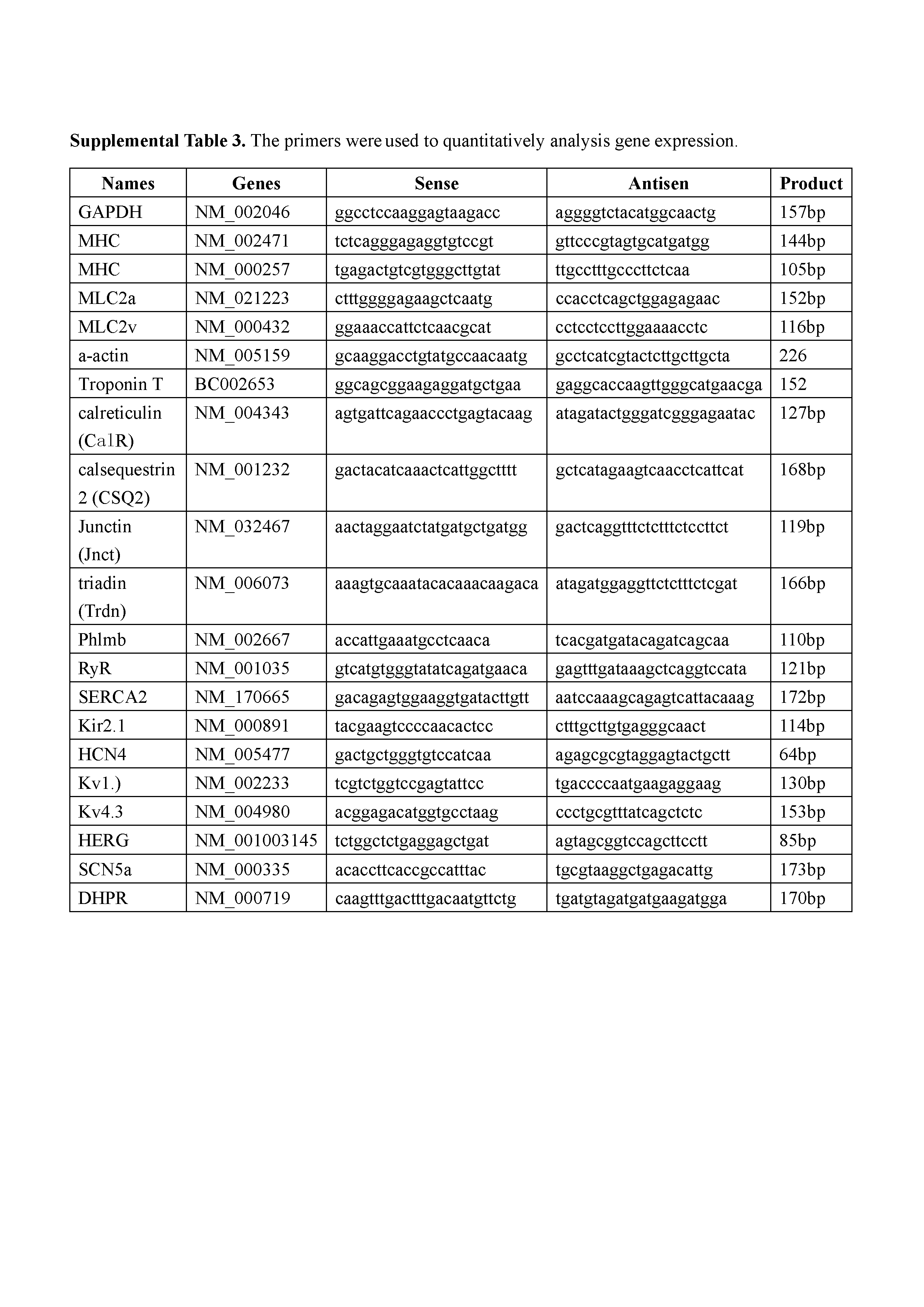

Supplement: Table S3 — The primers were used to quantitatively analysis gene expression. (TIFF) [file pone.0027417.s009.tif]
